# Evaluation of osteoporosis knowledge among adult Saudi Women in Tabuk, Saudi Arabia: A cross-sectional study

**DOI:** 10.1097/MD.0000000000048047

**Published:** 2026-03-13

**Authors:** Hamad S. Al Amer, Samah S. Zahran, Razan A. Al-balawi, Shahad H. Alhawiti, Raghad N. Albalawi, Alhanouf E. Albalawi, Leena K. Almashyakhi

**Affiliations:** a Department of Health Rehabilitation Sciences, Faculty of Applied Medical Sciences, University of Tabuk, Tabuk, Saudi Arabia; b Department of Musculoskeletal Disorders and Their Surgery, Faculty of Physical Therapy, Cairo University, Giza, Egypt.

**Keywords:** knowledge, osteoporosis, Saudi Arabia, women

## Abstract

Osteoporosis (OP) is more common in women than men. Evaluating women’s knowledge of the risk factors, signs, and symptoms of OP is important as this would be useful for developing proper preventive measures. Therefore, this study aimed to evaluate the level of OP knowledge of women in Tabuk, Saudi Arabia. In this cross-sectional study, 284 adult Saudi females completed a questionnaire that collected their demographic and health characteristics, as well as their level of OP knowledge, using the Osteoporosis Knowledge Assessment Tool. Data were analyzed using the chi-square test, *t*-test, and analysis of variance, with an alpha level of 0.05. The prevalence of OP was 6%. Approximately 18% of the sample had poor overall OP knowledge, whereas 77% and 4% had good and very good knowledge, respectively. Furthermore, the participants showed better knowledge of the symptoms and risk of fracture due to OP, followed by OP risk factors. However, they had less knowledge of the treatment availability and protective factors against OP. Several demographic factors, including age (middle adulthood vs early adulthood; *P* = .030 and vs old age; *P* = .033), education (illiterate vs general; *P* = .032, vs university; *P* = .023, and vs higher education; *P* = .002), and marital status (divorced vs single; *P* = .013, vs married; *P* = .044, widow vs single; *P* = .003, and vs married; *P* = .011), as well as employment status (*P* = .013), significantly affected the level of knowledge. Health-related factors, including diagnosis of OP (*P* = .008), family history of OP (*P* = .037), history of fracture (*P* = .026), and early menopause (*P* = .044), were also significantly associated with higher knowledge scores. Most Saudi women in Tabuk have a good overall level of knowledge about OP. Although they had better knowledge of OP symptoms and risk factors, they had less knowledge of the treatment availability and protective factors against OP, such as proper diet and physical activity. We recommend that more efforts be made to raise awareness and increase education regarding OP, especially among older and less-educated women.

## 1. Introduction

Osteoporosis (OP) is a skeletal disease that causes bone weakness and increases the vulnerability to fractures. Bone strength is fundamentally influenced by density and quality. A key feature of OP is the deterioration of the trabecular microarchitecture, which is characterized by decreased connectivity among trabecular elements and thinning of the cortical bone. This condition poses a serious health risk, particularly in older adults, and highlights the need for effective preventive and treatment strategies.^[1,2]^

Studies in Saudi Arabia (SA) have reported that up to 41% of adults have OP.^[3,4]^ In hospital-based settings, the prevalence is significantly higher, with estimates of 52.8% and 63.6% for women and men, respectively.^[5]^ Additionally, the incidence of femoral fractures due to OP is estimated to be approximately 5.81/1000 in the general population, with annual direct and indirect costs (e.g., productivity loss and caregiving expenses) of SR of 2.359 billion.^[6]^

Healthcare authorities classify OP as a public health issue owing to its association with an elevated risk of serious fractures. Nontraumatic fractures, which are the most prominent clinical signs of OP, lead to a decline in the overall quality of life, particularly in those with multiple fractures. A reduction in quality of life after fragility fracture can result from chronic pain, limitations in daily activities, or decreased mobility.^[7]^

The early diagnosis of OP is challenging and often identified only after the occurrence of fractures. Early assessment and treatment are critical to reduce the risk of OP fractures. Consequently, to effectively manage the disease, general awareness of the causes and risk factors needs to be increased. Aging, heavy drinking, smoking, family history of OP, lack of physical activity, inadequate calcium and vitamin D intake, and white ancestry are risk factors associated with the occurrence of OP.^[8]^ Studies have indicated that knowledge and understanding these risk factors could help prevent OP.^[9,10]^

A 2016 study evaluating OP knowledge in SA reported that older participants displayed better understanding than younger participants.^[11]^ Two studies in Riyadh highlighted the poor application of preventive measures among Saudi females.^[12,13]^ However, females attending family medicine clinics demonstrated good knowledge, although younger females were less knowledgeable, indicating the need for increased awareness.^[14]^ Another study in 2017 confirmed good overall knowledge among participants living in Riyadh, primarily acquired from relatives and physicians, emphasizing the necessity of educational programs in healthcare facilities to support OP prevention.^[15]^

To the best of our knowledge, no study has evaluated the level of OP knowledge among females in Tabuk, SA. This study aimed to evaluate the level of OP knowledge in a sample of Saudi women residing in Tabuk, SA, and to examine the effect of demographic and health characteristics on the level of knowledge.

## 2. Methods

### 2.1. Design

This study used a cross-sectional design to evaluate OP knowledge levels. A nonrandom convenience sample from Tabuk was selected for participation in this study.

### 2.2. Participants

Adult Saudi women from Tabuk’s local community aged 18 years and older who agreed to participate in the study were enrolled. The Raosoft calculator^[16]^ determined a minimum sample size of 270, with a confidence level of 90% and a margin of error of 5%, and estimated the population of adult Saudi females in Tabuk City in 2022 to be approximately 123,000 according to the General Authority for Statistics in SA.^[17]^ Therefore, a sample size of 300 was determined when considering missing responses. Participants recruitment started from January 2024 and for a duration of 2 months. All participants signed a written consent form before participating in the study, which was approved by the University of Tabuk’s Local Research Ethics Committee (approval number: UT-343-177-2024).

### 2.3. Instruments

A self-designed, 3-part questionnaire was used for data collection as follows: Part 1 collected demographic information and Part 2 collected health information. Part 3 includes the Osteoporosis Knowledge Assessment Tool (OKAT).^[18]^ The OKAT consists of 20 questions that assess the level of knowledge about OP, with response options of “True,” “False,” or “Don’t know.” One point is given for each correct answer and 0 points for each incorrect or “don’t know” answer. The maximum possible score was 20. For this study, the OKAT was translated into Arabic and evaluated for face and content validity by a group of ten health professional experts. All ten experts rated the translated tool items as valid and relevant (average face validity rating = 0.83; item-level content validity index = 0.70 to 1.00; average scale-level content validity index = 0.84). One item on alcohol consumption was removed from the translated version, as all experts unanimously decided that this item was irrelevant to the Saudi context. The remaining 19 items of the OKAT were divided into 4 subscales as follows: Subscale 1: knowledge about OP risk factors (7 questions: 3, 4, 5, 6, 7, 12, and 17); Subscale 2: knowledge about symptoms and risk of fracture due to OP (5 questions: 1, 2, 8, 9, and 11); Subscale 3: knowledge about OP treatment availability (2 questions: 18 and 19); and Subscale 4: knowledge about protective factors against OP (5 questions: 10, 13, 14, 15, and 16).^[14]^

### 2.4. Statistical analyses

Data pertaining to participants’ knowledge based on their answers to individual OKAT items, overall level of knowledge based on their OKAT total scores, level of knowledge on the OKAT subscales, and the effect of participants’ characteristics on their level of knowledge were statistically analyzed.

To determine participants’ knowledge of individual OKAT items, we calculated the percentage of participants who answered each item of the OKAT correctly compared to those who answered them incorrectly based on the individual OKAT items. The ratios were interpreted as follows: <25%, poor knowledge; 25% to >50%, fair knowledge; 50% to >75%, good knowledge; and ≥75%, very good knowledge. The significance of the differences between percentages was tested using the chi-square test. To determine the overall level of knowledge, OKAT total scores were classified as follows: ≤7, poor knowledge; 8 to 13, good knowledge; and ≥14, very good knowledge.^[14]^

To compare the participants’ knowledge levels of the OKAT subscales, their scores were first converted to percentages because the range of scores differed from 1 subscale to another. Repeated-measures analysis of variance (ANOVA) was performed to detect differences in knowledge among the subscales. Repeated-measures ANOVA was used as the subscale scores were derived from the same group of participants (i.e., within-subject measurements). Follow-up pairwise comparisons among OKAT subscale scores were performed using Fisher Least Significant Difference post hoc test following a significant repeated-measures ANOVA.

Finally, *t*-tests for independent samples were used to examine the impact of participant demographic and health characteristics on the level of OP knowledge. An alpha level of.05 was used in all statistical analyses conducted using SPSS for Windows version 25.0 (Chicago).

## 3. Results

### 3.1. Participants

A total of 300 questionnaires were distributed, and 284 responses were received, with a response rate of 94.7 %. Respondents’ demographic and health characteristics are presented in Table 1. Most participants were 18 to 39 years old (72.9%), single (48.2%), had a university education (53.2%), unemployed (66.2%), and had a monthly income of 10,000 SR or less (64.8%). Half of the 36 females who reported discontinuity of the menstrual cycle had early menopause. Furthermore, 19.4% of participants had a family history of OP, whereas 16.5% had a history of fracture. Of 284 females included in this study, 17 (6%) were diagnosed with OP.

**Table 1:** Characteristics of the participants (n = 284).

Characteristics		n	%
Age (year)	18–39 (early adulthood)	207	72.9
	40–59 (middle adulthood)	65	22.9
	≥ 60 (old age)	12	4.2
Marital status	Single	137	48.2
	Married	119	41.9
	Divorced	17	6
	Widow	11	3.9
Education	General	101	35.6
	University	151	53.2
	Higher education	15	5.3
	Illiterate	17	6
Employment status	Employed	96	33.8
	Unemployed	188	66.2
Monthly income (SR)	<10,000	184	64.8
	10,000–20,000	86	30.3
	>20,000	14	4.9
Monthly period	Continuous	248	87.3
	Discontinuous	36	12.7
Early menopause*	Yes	18	50
	No	18	50
Family history of OP	Yes	55	19.4
	No	229	80.6
History of fracture	Yes	47	16.5
	No	237	83.5
Diagnosed with OP	Yes	17	6
	No	267	94
Weight mean ± SD (kg)		61.3 ± 13.44	
Height mean ± SD (m)		1.6 ± 0.06	
Body mass index mean ± SD (kg/m^2^)		24.6 ± 4.95	

OP = osteoporosis, SD = standard deviation, SR = Saudi Riyal.

* n = 36.

### 3.2. Level of knowledge about OP

#### 3.2.1. Level of knowledge of individual OKAT items

Table 2 shows the percentage of participants who responded correctly to each item of the OKAT. The participants showed very good knowledge for 2 questions and good knowledge for 8 questions. However, they showed a fair level of knowledge for 5 questions and poor level of knowledge for 4 questions. Except for Item 4, the differences between the proportions of respondents who answered correctly and those who did not for each item were statistically significant.

**Table 2:** Knowledge level based on OKAT individual items (n = 284).

OKAT item	Correct answer	% of the correct answer	Knowledge level	*P*-value
Item 1	True	92.3	Very good	<.001*
Item 2	False	5.3	Poor	<.001*
Item 3	False	44.0	Fair	.044*
Item 4	False	53.5	Good	.235
Item 5	True	68.7	Good	<.001*
Item 6	True	17.6	Poor	<.001*
Item 7	True	81.7	Very good	<.001*
Item 8	True	59.9	Good	.001*
Item 9	True	34.5	Fair	<.001*
Item 10	False	17.3	Poor	<.001*
Item 11	True	73.9	Good	<.001*
Item 12	True	62	Good	<.001*
Item 13	True	67.3	Good	<.001*
Item 14	True	56.3	Good	.033*
Item 15	False	42.6	Fair	.013*
Item 16	True	25.0	Fair	<.001*
Item 17	False	8.1	Poor	<.001*
Item 18	True	25.7	Fair	<.001*
Item 19	False	56.3	Good	.033*

Significant differences were examined using Chi-square tests.

OKAT = osteoporosis knowledge assessment tool.

* Significant difference at α = 0.05.

#### 3.2.2. Overall level of knowledge

The mean OKAT score of the participants was 8.9 ± 2.52, ranging from 1 to 16 points. Based on these scores, 52 participants (18.3%) had poor overall knowledge (score ≤7), 220 participants (77.5%) had good knowledge (score 8–13), and 12 participants (4.2%) had very good knowledge (score ≥14).

#### 3.2.3. Level of knowledge of OKAT subscales

Table 3 shows the participants’ scores on the 4 OKAT subscales. The results of the 1-way ANOVA showed significant differences in the level of knowledge among the study samples regarding the obtained scores on these subscales (*P* <.001). As shown in Table 4, follow-up pairwise comparisons revealed that participants scored significantly higher on the symptoms and risk of fracture subscale (53.2%) compared to the risk factors subscale (47.9%; *P* <.001), the protective factors subscale (41.7%; *P* <.001), and the treatment availability subscale (41.0%; *P* <.001). The risk factors subscale score was also significantly higher than those of the protective factors (*P* <.001) and treatment availability (*P* <.001) subscales. However, there was no significant difference between scores on the protective factor and treatment availability subscales (*P* = .742).

**Table 3:** Mean scores of the participants on the OKAT subscales (n = 284).

Subscale	Points		Percentage			
	Range	Mean ± SD	Range	Mean ± SD	*F*	*P*-value
Risk factors (7 points)	0–6	3.4 ± 1.26	0–85.7	47.9 ± 17.93	20.8	<.001*
Symptoms and risk of fracture (5 points)	0–5	2.7 ± 1.06	0–100	53.2 ± 21.26		
Treatment availability (2 points)	0–2	0.8 ± 0.62	0–100	41.0 ± 31.15		
Protective factors (5 points)	0–5	2.1 ± 1.01	0–100	41.7 ± 20.10		

OKAT = osteoporosis knowledge assessment tool, SD = standard deviation.

* Significant at α = 0.05.

**Table 4:** Results of all pairwise comparisons for the OKAT subscale scores (n = 284).

Pairwise comparison	*t*	*P*-value
Risk factors versus symptoms and risk of fracture	3.65	<.001*
Risk factors versus treatment availability	3.55	<.001*
Risk factors versus protective factors	4.45	<.001*
Symptoms and risk of fracture versus treatment availability	5.84	<.001*
Symptoms and risk of fracture versus protective factors	7.08	<.001*
Treatment availability versus protective factors	0.33	.742

OKAT = osteoporosis knowledge assessment tool.

* Significant at α = 0.05.

#### 3.2.4. Effect of participant characteristics on level of knowledge

As shown in Table 5, participants with a family history of OP scored significantly higher on the risk factors subscale (*P* = .037). On the symptoms and risk of fracture subscale, illiterate women scored significantly lower than those with a general (*P* = .032), university (*P* = .023), or higher education (*P* = .002). Single and married women achieved higher scores than divorced (*P* = .013 and *P* = .044, respectively) and widowed women (*P* = .003 and *P* = .011, respectively). Additionally, females who were diagnosed with OP scored higher on this subscale than those who were not (*P* = .008).

**Table 5:** Effect of participants characteristics on level of knowledge scores (n = 284).

Characteristics	OKAT subscale score				Test results	
	Subgroup	M ± SD	Subgroup	M ± SD	*t*	*P*-value
Risk factors subscale						
Family history of OP	Yes	3.7 ± 1.14	No	3.3 ± 1.27	2.10	.037*
Symptoms and risk of fracture subscale						
Educational level	Illiterate	2.1 ± 1.03	General	2.7 ± 0.95	2.16	.032*
			University	2.7 ± 1.11	2.29	.023*
			Higher education	3.2 ± 1.08	3.06	.002*
Marital status	Single	2.8 ± 0.98	Married	2.7 ± 1.04	0.95	.342
			Divorced	2.1 ± 1.36	2.50	.013*
			Widow	1.8 ± 1.25	2.97	.003*
	Married	2.7 ± 1.04	Single	2.8 ± 0.98	0.95	.342
			Divorced	2.1 ± 1.36	2.02	.044*
			Widow	1.8 ± 1.25	2.57	.011*
Diagnosed with OP	Yes	2.7 ± 1.05	No	2.0 ± 1.12	2.66	.008*
Treatment availability subscale						
Early menopause†	Yes	1.1 ± 0.68	No	0.7 ± 0.59	2.10	.044*
Protective factors subscale						
Age	Middle adulthood	2.3 ± 0.89	Early adulthood	2.0 ± 1.03	2.18	.030*
			Old age	1.7 ± 1.98	2.15	.033*
Employment status	Employed	2.3 ± 0.92	Unemployed	2.0 ± 1.03	2.50	.013*
History of fracture	Yes	2.4 ± 0.85	No	2.0 ± 1.02	2.24	.026*

M = mean, OKAT = osteoporosis knowledge assessment tool, OP = osteoporosis, SD = standard deviation.

† n = 36.

* Significant at α = 0.05.

Regarding the treatment availability subscale, women with early menopause had higher mean scores than those without (*P* = .044). Finally, females with a history of fractures and employed women had higher mean scores on the protective factor subscale than their counterparts (*P* = .026 and *P* = .013, respectively). Furthermore, middle-aged women obtained greater scores on the same subscale than did young (*P* = .030) and older females (*P* = .033).

## 4. Discussion

This study aimed to assess the level of OP knowledge among Saudi women living in Tabuk, SA, and examine the impact of their demographic and health characteristics on this knowledge. The observed prevalence of OP in this population was comparable to findings reported in other Saudi cities, such as Riyadh (5%),^[15]^ suggesting a consistent trend across different regions.

In this study, we identified poor knowledge related to physical activities that improve bone health, rate of bone loss after menopause, at-risk race, and disease symptoms. This highlights the crucial role of healthcare providers in raising public awareness about OP, as professionals can only effectively communicate specialized information. However, participants showed greater awareness of the fracture risks associated with OP and the role of falls, suggesting some existing understanding of OP consequences.

Our results on the level of OP knowledge among women in Tabuk are comparable to those of another study conducted among Saudi females visiting family medicine clinics in Riyadh, SA^[14]^ where the mean score was 66%, and 59.8% of the participants achieved a good score on the questionnaire. Another study among Saudi women in Riyadh reported that, while 31% had poor knowledge, the majority of the individuals had good knowledge at 69%.^[15]^ Additionally, a study involving female students recruited from 4 Saudi universities reported that 57.7% had good or high overall knowledge.^[19]^ A study of adult females in Majmaah City, SA, revealed that half of the participants had a fair level of knowledge about OP.^[20]^ Similarly, a study involving adult women in Cairo, Egypt reported an average total OKAT score of 10.5, with a total knowledge percentage score of 52.3%; a score of 60% or above was obtained by less than half of the women (41.5%).^[21]^ Women from Qatar had an overall knowledge level of 61.4%.^[22]^ A recent systematic review in China reported that the level of OP knowledge and awareness in the Chinese population was moderate to low.^[23]^

Understanding the relationship between aging, fractures, and OP is critical for preserving bone health. Early identification through bone mineral density assessments combined with preventive strategies, such as optimized nutrition, regular physical activity, and pharmacological interventions, can significantly reduce the fracture risk and mitigate the risk of associated functional impairments.^[24]^ In our study, participants showed more knowledge concerning the symptoms, risk of fracture, and aging associated with OP, similar to Saudi females in Riyadh, as the percentage of correct answers was highest for awareness of symptoms and fracture risk due to OP (83.6%).^[14]^

In this study, participants’ understanding of OP appeared to be influenced by their educational background, personal health history, and social circumstances. Educated individuals may have better access to information on OP, which could enhance their awareness of symptoms and risks. Single women in our study were highly educated, which may explain their higher awareness than divorced and widowed women. Our findings are in agreement with those of a study conducted in Majmaah City^[20]^ wherein married participants and those holding a bachelor’s degree exhibited the highest levels of knowledge in comparison with other groups. Additionally, a study in Qatar^[22]^ reported that younger, less-educated women who had never married demonstrated the lowest level of knowledge.

Understanding the risk factors of OP is essential for preventing and effectively managing the disease. By being aware of these factors, individuals can take preventive action and healthcare providers can design health plans that minimize bone loss and decrease the risk of fractures. In our study, the awareness of OP risk factors was relatively strong, particularly regarding the importance of falls in causing fractures. However, knowledge of the rate of bone loss after menopause was limited. Awareness of family history as a risk factor was also notable and appeared higher than in previous studies, exceeding the 56.1% reported by Alqahtani et al,^[14]^ the 22% reported by Alshahrani et al^[25]^ in Riyadh, and the 58.6% reported by Abumunaser et al^[26]^ in Jeddah, SA.

This association between family history of OP and increased awareness of the risk factor observed in this study may be attributed to greater exposure to information among individuals with affected relatives. Such individuals are likely to be more exposed to information about the disease through healthcare interactions, family discussions, or personal experiences. These factors may lead to a deeper understanding of OP and its risk factors in individuals with affected relatives than in those without a family history.

The lowest knowledge were observed for the preventive factors and treatment availability. Although the participants demonstrated good knowledge of various calcium sources and adequate daily calcium intake, only a small proportion recognized their role in preventing OP. Moreover, awareness of dietary calcium and alternative sources such as sardines and broccoli was lower than what has been reported by Alqahtani et al,^[14]^ who found awareness levels of 83% for recommended intake and 91% for alternative calcium sources. However, our findings are comparable to those of Alshareef et al^[27]^ who reported scores of 68% and 57%, respectively, among Saudi female college students.

A possible limitation of this study is that only Saudi females from Tabuk City were recruited. Other cities in the Tabuk region were excluded. Therefore, the results of the study cannot be generalized on the whole Tabuk region. Additionally, the use of a convenience sampling method may further limit the external validity of the results. Future research should consider using random sampling and include participants from other cities and nationalities to provide a broader understanding of OP knowledge across the Tabuk region. Another limitation is the exclusion of alcohol consumption items from the OKAT, as this question could be irrelevant to the Saudi community. Nevertheless, this could have had a minimal effect on the results, as the total score of the OKAT was averaged for 19 rather than 20 questions.

## 5. Conclusions

This study found that most Saudi women in Tabuk had a generally good level of knowledge about OP, particularly regarding its symptoms and risk factors. However, awareness of treatment options and protective measures, such as proper diet and physical activity, was limited. Certain demographic and health-related factors, such as employment, history of fractures, and educational background, appeared to be associated with higher knowledge levels. These results highlight the need for targeted educational interventions to enhance OP prevention strategies across different demographic groups, especially among older and less-educated women.

## Acknowledgments

The authors would like to acknowledge the participants who volunteered for this study.

## Author contributions

**Conceptualization:** Hamad S. Al Amer, Samah S. Zahran, Razan A. Al-balawi.

**Data curation:** Hamad S. Al Amer, Samah S. Zahran, Shahad H. Alhawiti.

**Formal analysis:** Hamad S. Al Amer, Samah S. Zahran.

**Investigation:** Hamad S. Al Amer, Razan A. Al-balawi, Shahad H. Alhawiti, Raghad N. Albalawi, Alhanouf E. Albalawi, Leena K. Almashyakhi.

**Methodology:** Hamad S. Al Amer, Samah S. Zahran.

**Project administration:** Hamad S. Al Amer.

**Supervision:** Hamad S. Al Amer, Samah S. Zahran.

**Validation:** Hamad S. Al Amer, Samah S. Zahran, Razan A. Al-balawi, Raghad N. Albalawi.

**Writing – original draft:** Hamad S. Al Amer, Samah S. Zahran, Razan A. Al-balawi, Shahad H. Alhawiti, Raghad N. Albalawi, Alhanouf E. Albalawi, Leena K. Almashyakhi.

**Writing – review & editing:** Hamad S. Al Amer.
